# Global human influence maps reveal clear opportunities in conserving Earth’s remaining intact terrestrial ecosystems

**DOI:** 10.1111/gcb.15109

**Published:** 2020-06-05

**Authors:** Jason Riggio, Jonathan E. M. Baillie, Steven Brumby, Erle Ellis, Christina M. Kennedy, James R. Oakleaf, Alex Tait, Therese Tepe, David M. Theobald, Oscar Venter, James E. M. Watson, Andrew P. Jacobson

**Affiliations:** ^1^ National Geographic Society Washington DC USA; ^2^ Department of Wildlife, Fish and Conservation Biology Museum of Wildlife and Fish Biology University of California, Davis Davis CA USA; ^3^ Department of Geography and Environmental Systems University of Maryland Baltimore County MD USA; ^4^ Global Lands Program The Nature Conservancy Fort Collins CO USA; ^5^ Conservation Planning Technologies Fort Collins CO USA; ^6^ Natural Resource and Environmental Studies Institute University of Northern British Columbia Prince George BC Canada; ^7^ School of Earth and Environmental Science The University of Queensland Brisbane Qld Australia; ^8^ Global Conservation Wildlife Conservation Society Bronx NY USA; ^9^ Department of Environment and Sustainability Catawba College Salisbury NC USA

**Keywords:** Anthromes, conservation targets, Convention on Biological Diversity, Global Human Modification, habitat intactness, Half‐Earth, Human Footprint, human influence, Low Impact Areas, spatial conservation prioritization

## Abstract

Leading up to the Convention on Biological Diversity Conference of the Parties 15, there is momentum around setting bold conservation targets. Yet, it remains unclear how much of Earth's land area remains without significant human influence and where this land is located. We compare four recent global maps of human influences across Earth's land, Anthromes, Global Human Modification, Human Footprint and Low Impact Areas, to answer these questions. Despite using various methodologies and data, these different spatial assessments independently estimate similar percentages of the Earth's terrestrial surface as having very low (20%–34%) and low (48%–56%) human influence. Three out of four spatial assessments agree on 46% of the non‐permanent ice‐ or snow‐covered land as having low human influence. However, much of the very low and low influence portions of the planet are comprised of cold (e.g., boreal forests, montane grasslands and tundra) or arid (e.g., deserts) landscapes. Only four biomes (boreal forests, deserts, temperate coniferous forests and tundra) have a majority of datasets agreeing that at least half of their area has very low human influence. More concerning, <1% of temperate grasslands, tropical coniferous forests and tropical dry forests have very low human influence across most datasets, and tropical grasslands, mangroves and montane grasslands also have <1% of land identified as very low influence across *all* datasets. These findings suggest that about half of Earth's terrestrial surface has relatively low human influence and offers opportunities for proactive conservation actions to retain the last intact ecosystems on the planet. However, though the relative abundance of ecosystem areas with low human influence varies widely by biome, conserving these last intact areas should be a high priority before they are completely lost.

## Graphical abstract

Leading up to the Convention on Biological Diversity Conference of the Parties 15, there is momentum around setting bold conservation targets. Yet it remains unclear how much of Earth's land area remains without significant human influence. We compare four recent global maps of human influences (Anthromes, Global Human Modification, Human Footprint, and Low Impact Areas) and find that about half of Earth's terrestrial surface has relatively low human influence. These findings offer opportunities for proactive conservation actions to retain the last intact ecosystems on the planet, although the relative abundance of ecosystem areas with low human influence varies widely by biome.

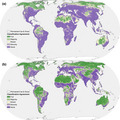

## 1 INTRODUCTION

Ecosystems that have low human influence are vital contributors to human well‐being (Díaz et al., 2018), including providing ecosystem services (e.g., clean water and flood control, carbon storage and pollination; Watson, Venter, et al., 2018), buffering against climate change (Martin & Watson, 2016) and housing biodiversity (Di Marco, Ferrier, Harwood, Hoskins, & Watson, 2019). These so‐called ‘wild’ or ‘wilderness’ areas are also important as places of spiritual and mental renewal, exploration and wonder (Ewert, Overholt, Voight, & Wang, 2011) and serve many local communities by sustaining long‐term cultural connections with these places (Garnett et al., 2018; Watson, Evans, et al., 2018). However, as human populations and economies have expanded, so too have human influences on natural environments (Venter et al., 2016). These human‐influenced environments, such as agricultural, forestry and urban areas, can still retain or be managed to support some elements of biodiversity and be important areas in providing ecosystem services or recreation (Ellis, 2019; Locke et al., 2019). Indeed, land sharing or wildlife‐friendly agriculture can help protect biodiversity (Green, Cornell, Scharlemann, & Balmford, 2005; Kremen & Merenlender, 2018), selectively logged forests can retain many, but not all, of their ecosystem services (Edwards, Tobias, Sheil, Meijaard, & Laurance, 2014), and urban areas can be hotpots for pollinators (Baldock et al., 2019). Nevertheless, human societies now consume a quarter of net primary productivity (Krausmann et al., 2013), and while the importance of intact, natural systems is increasingly recognized, they are being rapidly eroded (Oakleaf et al., 2015; Watson, Venter, et al., 2018). These losses persist even as countries have committed in the Convention on Biological Diversity (CBD) to dramatically decrease the rate of loss of natural habitats and to significantly reduce their degradation and fragmentation by 2020 (e.g., Aichi Target 5; CBD & UNEP, 2010).

Although nearly 20% of the terrestrial surface of the planet is classified as built‐up or cropland (Defourny et al., 2017), only 15% of Earth's land surface is formally under protection (UNEP‐WCMC, IUCN, & NGS, 2019). In addition, Earth's remaining intact ecosystems outside the protected area estate (and even within them, Jones et al., 2018) are being rapidly eroded (Kennedy, Oakleaf, Theobald, Baruch‐Mordo, & Kiesecker, 2019; Watson, Shanahan, et al., 2016). Nations will set new conservation targets at the CBD Conference of the Parties 15. Leading up to the convention, there is momentum behind setting ambitious targets of conserving at least 50% of the Earth's surface by 2050 (e.g., Baillie & Zhang, 2018; Dinerstein et al., 2017, 2019; Locke, 2013; Wilson, 2016). To make these targets effective, evidence‐based ecological assessments (Pimm, Jenkins, & Li, 2018; Watson & Venter, 2017) are needed to answer a key question: how much of Earth's terrestrial surface remains without intensive human use is currently left to conserve?

Initial efforts to map human influence globally started in the 1980s with a focus on identifying wilderness (i.e., areas free from human alteration; e.g., McCloskey & Spalding, 1989) and progressed through the 1990s (e.g., Hannah, Lohse, Hutchinson, Carr, & Lankerani, 1994; Lesslie, 1998) and 2000s (e.g., Mittermeier et al., 2002). These first maps, while revolutionary, were crude due to both data and computing limitations (Watson & Venter, 2019). The Human Footprint index was a significant step forward in mapping human pressures across the world's terrestrial lands (Sanderson et al., 2002). Human Footprint combined globally consistent digital data of known pressures on biodiversity (e.g., human population and cropland) in a geographic information system to generate a score that enabled a high‐resolution map (1 km) of human pressure, and consequently, a ‘last of the wild’ map. An important conceptual step in the mapping progression was the recognition that human and biological systems are intertwined and should be analyzed together, hence the delineation and categorization of Anthropogenic Biomes, or Anthromes (Ellis, Goldewijk, Siebert, Lightman, & Ramankutty, 2010; Ellis & Ramankutty, 2008). Human populations and land use land cover data were combined with vegetation data to identify more than 15 categories of various mixes of human uses with ecosystems (e.g., Urban, Residential Irrigated Cropland and Residential Rangelands). Both of these datasets have been updated and improved since their initial release (Goldewijk, Beusen, Doelman, & Stehfest, 2017; Venter et al., 2016), with the Human Footprint having been applied to assessments of global wilderness loss (Watson, Shanahan, et al., 2016), determining and predicting mammal species extinction risk (Di Marco, Venter, Possingham, & Watson, 2018), change in animal movement and behavior (Kühl et al., 2019; Tucker et al., 2018), global protected area effectiveness (Jones et al., 2018) and nations progress towards CBD targets (Watson, Jones, et al., 2016).

More recently, two new global human influence datasets have been developed. The Global Human Modification map (Kennedy, Oakleaf, Theobald, Baruch‐Mordo, & Kiesecker, 2018) that measures the spatial extent of 13 anthropogenic stressors and their estimated intensities of influence and produces a continuous 0–1 metric of the ecological condition of land. While it is mapped at a resolution of 1 km, much of the input data is at finer resolution and reflect recent land condition (median date 2016). It accounts for the proportion of each grid cell covered by the stressor and multiplies it by an intensity value based on ‘emergy’ measures of human‐induced impacts on biological, chemical and physical processes of lands (Kennedy et al., 2019). In addition, a new map of Low Impact Areas identifies landscapes with low human densities and impacts and not primarily managed for human needs (Jacobson, Riggio, Tait, & Baillie, 2019). These areas are categorized at two thresholds as either Very Low Impact or Low Impact Areas. They result from a categorical process that starts with the entire globe as low impact and then excises areas that are primarily managed or modified for human use at a 1 km resolution. If a cell has any urban or cropland extent, night‐time lights, or anthropogenic forest cover change, then it was no longer considered low impact. Impacts based on human population and livestock density are scaled by aridity such that higher densities are required to move a cell from low impact to non‐low impact in more humid environments (Jacobson et al., 2019).

In this paper, we compare four key global human influence datasets: Anthromes, Global Human Modification, Human Footprint and Low Impact Areas. We build off previous efforts that have compared agreement between geospatial datasets, such as degraded lands (Gibbs & Salmon, 2015), urban areas (Potere & Schneider, 2007), land cover classes (Klein Goldewijk & Ramankutty, 2004; Tuanmu & Jetz, 2014) and human influence maps based on original values and classifications (Kennedy et al., 2019). Here, for the first time, we present a comparison of the agreement and disagreement in areas mapped as low human influence globally to answer two critical questions at the foundation of forthcoming ambitious targets: Is 50% of land left with little human influence on it, which protected area expansion can proactively conserve and where is it? Summary statistics identify the levels of agreement and disagreement between the four data layers both globally and across different biomes. In producing our congruency map, we identify further recommendations that could lead to improvements in future mapping efforts.

## 2 METHODS

For each human influence dataset, we produced two binary outputs—very low human influence and low human influence—according to the native dataset definitions (Table 1).

**TABLE 1:** Input dataset thresholds for low and very low human influence

Human influence dataset	Very low influence threshold	Low influence threshold
Anthromes	‘Wildlands’ (61, 62 and 63)	43, 53, 54, 61, 62 and 63
Global Human Modification	0–0.01	0–0.1
Human Footprint	0	0–3
Low Impact Areas	Categorical	Categorical

For the Anthromes dataset, we grouped the wild woodlands, wild treeless and barren lands, and wild ice classes into the very low human influence threshold—wildlands being defined as ‘lands without human populations or substantial land use’ (Ellis et al., 2010). We included these in addition to remote rangeland, semi‐natural remote woodlands, and semi‐natural treeless and barren lands to delineate the low human influence threshold. In Kennedy et al. (2019), low modification areas were classified as areas with <10% of human modification per 1 km^2^ and had median HM values on the lower half of the distribution globally (HM ≤ 0.10) and, on average, two overlapping human stressors. For this analysis, we assigned very low modification to 1 km^2^ cells with <1% of mapped human modification (HM ≤ 0.01), which were areas with very small proportions of only one low‐intensity human stressor and coincided with the common level of modification detected in strict protected areas (i.e., median HM = 0.008 in IUCN Ia & Ib protected areas). We considered areas having no pressure (i.e., value = 0) in the Human Footprint dataset to have very low human influence (Venter et al., 2016), while areas having low pressure (i.e., value ≤ 3), indicating ‘land which is predominantly free of permanent infrastructure, but may hold sparse human populations’ (Allan et al., 2017), as having low human influence. Finally, the Low Impact Areas dataset defines two categorical thresholds—low impact and very low impact. We used their low impact class (‘landscapes that currently have low human density and impacts and are not primarily managed for human needs’) for the low human influence threshold (Jacobson et al., 2019). For the very low human influence threshold, we used their very low impact class, which reduces the human population and livestock density thresholds to <1 per 1 km^2^ and excludes all raster cells containing roads.

Once the categories were set for each dataset, we standardized the projection and cell size for comparison purposes. We also identified the land grid cells that each dataset had in common because each had slightly different land/water boundaries. As the Global Human Modification and Human Footprint layers are natively set to 1 km raster cell resolution (Mollweide equal‐area projection), we used this as our comparison point, re‐projecting the Low Impact Areas dataset (from World Eckert IV) and resampling Anthromes (from ~5 km resolution) to meet this resolution and projection. We then overlaid all datasets together and used the resulting layer as a mask so that all four datasets were clipped to the same coastline, interior water, and permanent ice and snow boundaries, resulting in the same number of terrestrial cells for each dataset (128,207,944 cells). Fundamentally, this means that most of the inland water bodies, and areas of permanent ice and snow, were excluded from the analysis.

We first compared the standardized input datasets based on the aggregate percentage of the terrestrial surface of the world each classified as being in very low and low human influence. We then quantified how strongly correlated the binary spatial outputs from each dataset were by calculating pairwise Jaccard similarity coefficients (Jaccard, 1912). Jaccard similarity measures the size of the intersection between datasets (e.g., number of cells that both datasets classified as having very low human influence), divided by the size of the union of the datasets (e.g., number of cells that either dataset has classified as having very low human influence) to provide a statistic describing classification similarity between binary datasets. Values can range from 0 (no similarity) to 1 (identical datasets) and are presented as percent similarity. Therefore, we only considered the similarity between datasets in their classification of very low and low human influence cells.

To assess the aggregate percentage of the world classified as either low or very low influence by the input datasets, we divided the number of cells in either classification by the total number of land cells (see above). We then evaluated each cell as the number of times it was identified in the classification. This produced two comparison datasets. A value of 4 indicating full agreement with the low influence classification and a value of 0 indicating agreement in non‐low influence classification, while a value of 2 indicates greatest disagreement between datasets (i.e., an even split between very low influence and non‐low influence classifications). Intermediate values of 1 and 3 indicate a minority (one of the four) and majority (three of the four) of datasets classifying the cell as low influence, respectively. Using these overlay datasets, we calculated the overall spatial agreement for the binary classification of very low human influence and low human influence across all (values of 0 and 4) and the majority (3 out of 4) of datasets (values of 0, 1, 3 and 4). Similarly, we calculated these agreement statistics between these datasets at a biome level, categorizing cells by biome using the Ecoregions 2017 dataset (Dinerstein et al., 2017).

Finally, we calculated pairwise Jaccard distance (the inverse of Jaccard similarity; Levandowsky & Winter, 1971) between each human influence dataset to determine which biomes (Dinerstein et al., 2017; Ecoregions, 2017) have the greatest disagreement in their classification of very low and low human influence. We finally calculated the mean of the pairwise Jaccard distance values across all pairwise comparisons to provide a single value of dissimilarity in the classification of very low and low human influence for each biome.

## 3 RESULTS

**FIGURE 1:**
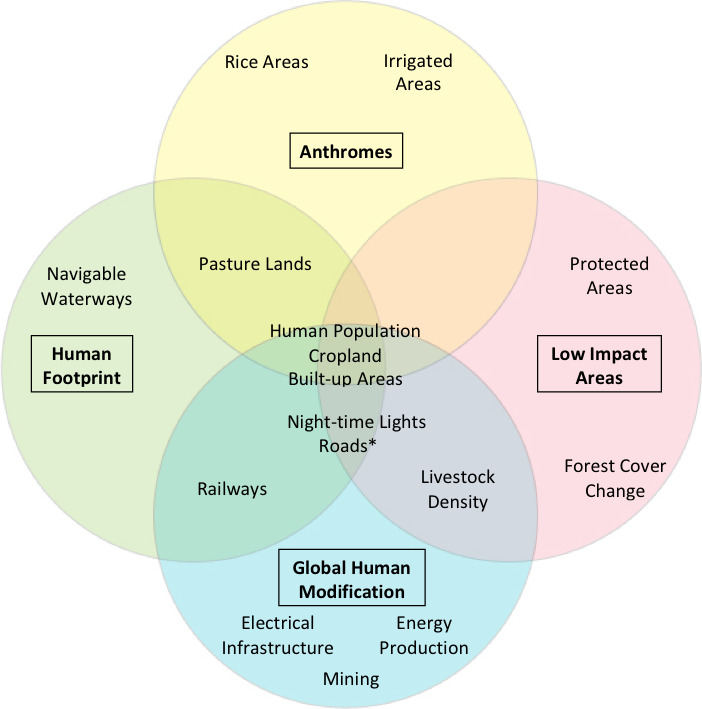
Venn diagram showing the various overlapping types of human stressors used in the Anthromes, Global Human Modification, Human Footprint and Low Impact Areas datasets. *Roads used to classify areas of very low human influence in the Low Impact Areas dataset

Each of the four human influence datasets uses their own unique definition, methodological approach and data inputs to identify areas of lower and higher ‘human influence’ (Table 2). Aside from the similarities and differences in definition and process, there is partial overlap in the data inputs (i.e., human stressors) used to produce these four datasets (Figure 1). All integrate spatial data on human population density, cropland and built‐up areas. However, each dataset considers unique human stressors: Anthromes uses rice and irrigated area as input layers; Global Human Modification incorporates electrical infrastructure, energy production and mining; Human Footprint includes access due to navigable waterways and coastlines; and Low Impact Areas uses forest cover change and strictly protected areas (Figure 1). Even when there is similarity in stressor type across the four datasets, there are few overlaps in the specific input dataset used for the stressor and how they were spatially mapped (Table S1; Table 2).

**TABLE 2:** Comparison of the methods used in creating the four global human influence datasets and their outputs

	Anthromes	Global Human Modification	Human Footprint	Low Impact Areas
Resolution	~5 km	1 km	1 km	1 km
Data year	2015	2016	2009	2015
Type	Categorical	Continuous	Ordinal	Categorical
Scaling	6 groups; 19 classes	0–1 (low to high)	0–50 (low to high)	3 classes
Definition	Human biomes—‘the globally significant ecological patterns created by sustained interactions between humans and ecosystems’	Ecological condition of lands based on the spatial extent and intensity of human activities	Cumulative human pressure on the environment	Landscapes with low human densities and impacts, and not primarily managed for human needs
Primary stressor datasets	6 (human population density, built‐up area, cropland, rice area, irrigated area, pasture)	13 (human population density, built‐up area, cropland, livestock, major roads, minor roads, two tracks, railroads, mines, oil wells, wind turbines, power lines, night‐time lights)	8 (human population density, built‐up area, cropland, pasture, major roads, railroads, navigable rivers, night‐time lights)	7 (human population density, built‐up area, cropland, livestock, forest cover change, roads [in very low impact class], night‐time lights)
Calculation of spatial extent	Classifications based on proportion of total area experiencing the stressor	Determined the proportion modified by each stressor per 1 km^2^ area (values ranged from 0 to 1)	Treated each stressor layer as present or absent	Treated each stressor layer as present or absent
Indirect effects due to human access	N/A	N/A	Applied a distance decay effect of for roads, navigable waterways and coastlines	N/A
Stressor weighting	N/A	Spatial extent × intensity value, continuous from 0 to 1	Assigned pressure scores from 0 to 10	Equal
Cumulative score	N/A	Applied fuzzy sum algorithm	Summation of cell values	N/A

The aggregate percentages mapped as very low and low human influence are similar across the four datasets at a global scale (Table 3). On average, just over 50% of the world is classified as low influence (48%–56%), while roughly a quarter of the planet's ice‐free terrestrial surface is considered very low influence (20%–34%).

**TABLE 3:** Percentage of the world classified as either low or very low human influence by the input datasets

Human influence dataset	Very low influence (%)	Low influence (%)
Anthromes	24.5	53.6
Global Human Modification	19.8	47.8
Human Footprint	25.3	49.2
Low Impact Areas	33.8	56.2
Average	25.9	51.7

All pairwise comparisons between the input datasets for both the very low and low human influence thresholds show greater than 50% similarity in classification with substantially higher similarity at the low compared to very low human influence level (Table 4). The Anthromes and Human Footprint datasets are the most similar for the very low influence threshold (57%; Table 4a), while the Anthromes and Global Human Modification datasets are the most similar in their classification of low human influence (73%; Table 4b). Conversely, the Anthromes and Global Human Modification datasets are the least similar for the very low influence threshold (52%; Table 4a), while the Human Footprint and Low Impact Areas datasets are the least similar in their classification of low human influence (65%; Table 4b). However, similarity in classifying land having very low and low human influence varies widely by biome (Table S2). For example, Global Human Modification and Human Footprint have less than 1% similarity in their classification in very low human impact in tropical dry forests, whereas Anthromes and Low Impact Areas have 98% similarity in their classification in low human impact in tundra.

**TABLE 4:** Pairwise Jaccard similarity coefficients for the input datasets classification of (A) very low and (B) low human influence

(A) Very low human influence	Anthromes	Global Human Modification	Human Footprint	Low Impact Areas
Anthromes				
Global Human Modification	52%			
Human Footprint	57%	53%		
Low Impact Areas	54%	55%	56%	

(B) Low human influence	Anthromes	Global Human Modification	Human Footprint	Low Impact Areas
Anthromes				
Global Human Modification	73%			
Human Footprint	68%	71%		
Low Impact Areas	68%	72%	65%	

**FIGURE 2:**
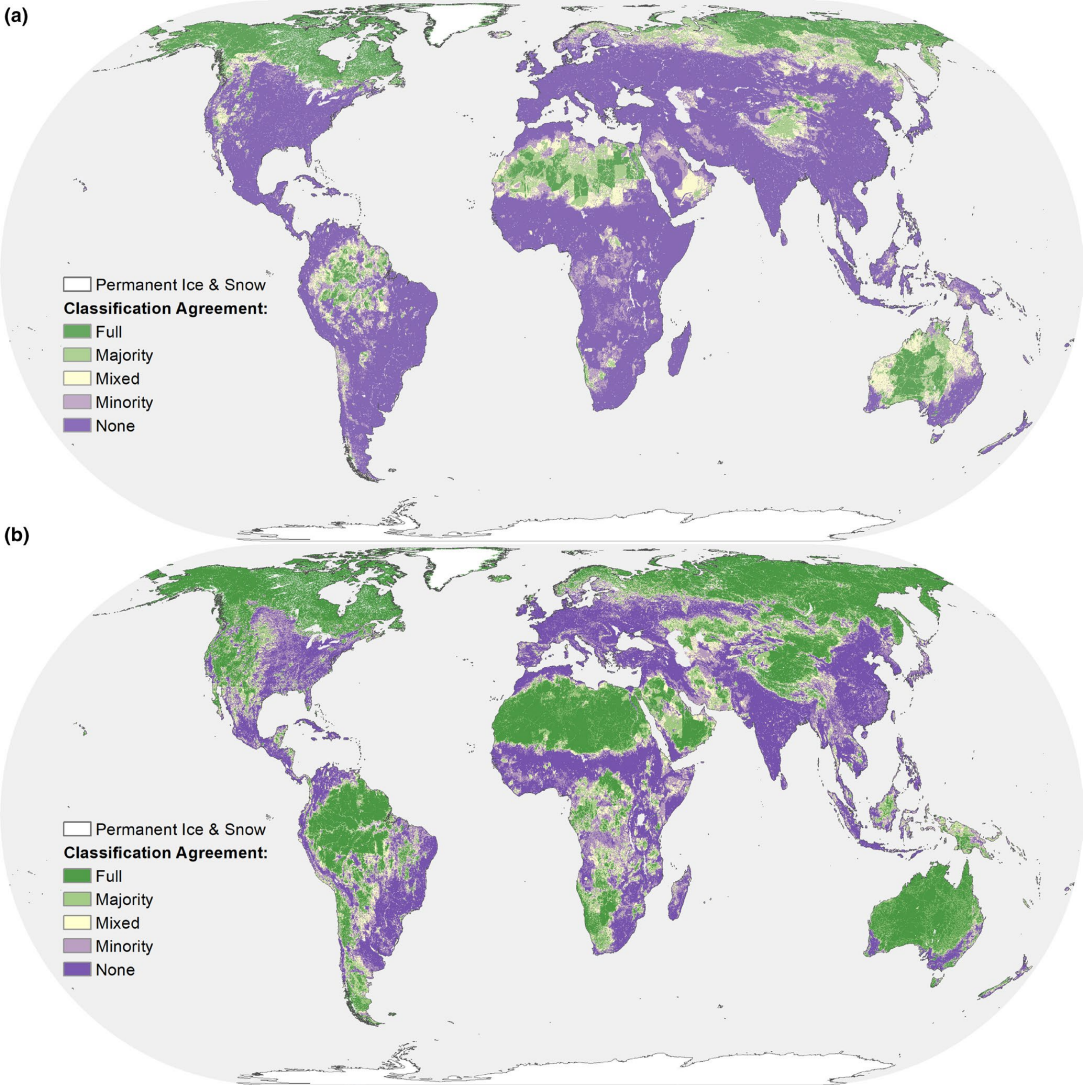
Maps showing the level of agreement between the four input datasets classification of (a) very low or (b) low human influence. ‘Full’ indicates all four datasets are in full agreement and all identify that cell as low (or very low) human influence, while ‘none’ indicates zero of the datasets identify that cell as low (or very low) human influence. ‘Majority’ reference areas where three out of the four, ‘Mixed’ two out of four and ‘Minority’ one out of four datasets identify that cell as low (or very low) human influence

**FIGURE 3:**
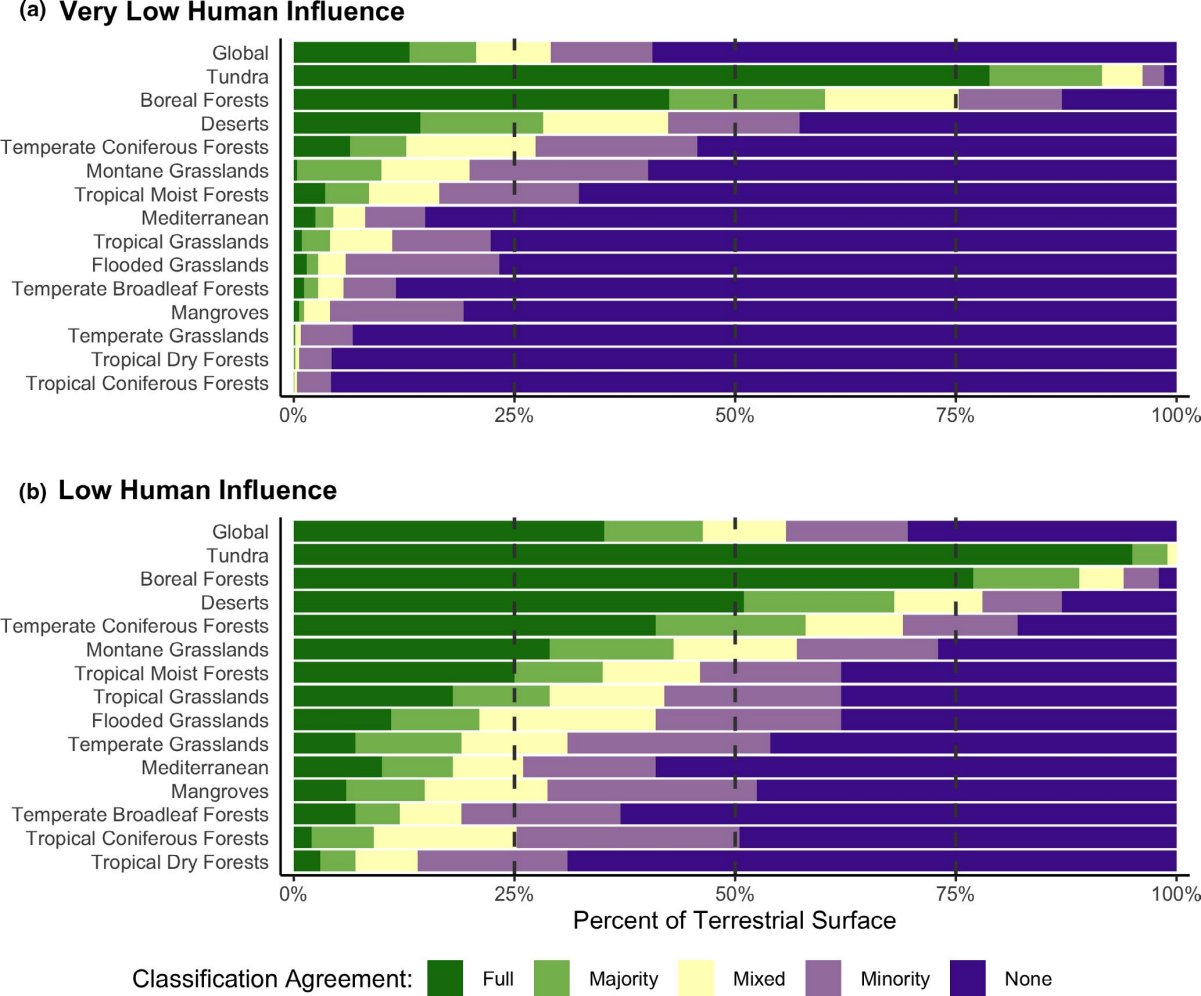
Overall percent agreement between the four input datasets classification of (a) very low or (b) low human influence of the terrestrial surface of the world and classified by biome. ‘Full’ indicates all four datasets are in full agreement and all identify that cell as low (or very low) human influence, while ‘none’ indicates zero of the datasets identify that cell as low (or very low) human influence. ‘Majority’ reference areas where three out of the four, ‘Mixed’ two out of four and ‘Minority’ one out of four datasets identify that cell as low (or very low) human influence

Much of the very low (Figure 2a; Figure S1a) and low influence (Figure 2b; Figure S2b) portions of the planet are comprised of cold (e.g., boreal forests, montane grasslands and tundra) or arid landscapes (i.e., deserts; Figure 3; Table S4). Encouragingly, however, a substantial portion of the Amazon Basin also contains a larger area of agreement for very low and low human influence.

Similarly, despite different objectives, input layers and methodologies, the overlay of the four input datasets shows substantial agreement at the coarse global scale for these binary classifications of human influence (Figure 3; Table S3). All four layers agree completely on just over a third of the planet's terrestrial surface as areas of low human influence (35%). While overall agreement of very low human influence is only 13%, the majority of datasets agree on 21% of the world as having very low influence. Overall, less than 10% of the planet has ‘Mixed’ classification results for both influence thresholds. These areas are likely the most challenging to assess for human influence.

Only two biomes (tundra and boreal forests) have a majority of datasets agreeing that at least half of their area has very low human influence (Figure 3a), while deserts and temperate coniferous forests have a majority of datasets agreeing that at least half of their area has low human influence (Figure 3b).

Worrisomely, <1% of temperate grasslands, tropical coniferous forests and tropical dry forests have very low human influence by the majority of datasets; and when considering full agreement, tropical grasslands, mangroves and montane grasslands also have <1% land identified as very low influence (Figure 3a).

When looking at average spatial classification agreement on a per biome basis, temperate coniferous forests, tropical dry forests, temperate grasslands and mangroves have the greatest disagreement at both the very low and low human influence thresholds (Table 5; Figure S2). For both influence thresholds, tundra, boreal forests and deserts have the greatest spatial agreement (Table 5; Figure S2).

**TABLE 5:** Average pairwise Jaccard distances (percent dissimilarity) for the input datasets classification of (A) very low and (B) low human influence per biome type

(A) Biome name	Very low influence (%)	(B) Biome name	Low influence
Tundra	11.4	Tundra	2.9
Boreal forests	31.4	Boreal forests	11.4
Deserts	50.7	Deserts	24.4
Mediterranean	62.1	Temperate coniferous forests	30.4
Temperate coniferous forests	64.5	Montane grasslands	38.1
Tropical moist forests	68.3	Tropical moist forests	39.1
Temperate broadleaf forests	69.6	Tropical grasslands	48.0
Tropical grasslands	77.4	Mediterranean	51.2
Montane grasslands	78.4	Temperate broadleaf forests	57.0
Flooded grasslands	79.3	Flooded grasslands	60.5
Mangroves	85.1	Temperate grasslands	60.6
Temperate grasslands	93.4	Mangroves	66.8
Tropical dry forests	94.3	Tropical dry forests	72.0
Tropical coniferous forests	96.3	Tropical coniferous forests	75.0

## 4 DISCUSSION

Despite varying input human stressor layers and methodologies employed by maps of Anthromes, Global Human Modification, Human Footprint and Low Impact Areas, the percentage of the terrestrial surface of the Earth that has very low and low human influence was found to be similar at global scales: 48%–56% low influence and 20%–34% very low influence (Table 3). Indeed, all pairwise comparisons between the input datasets for both the very low and low human influence thresholds show >50% similarity in spatial classification (Table 4). Furthermore, the majority of datasets agree on 46% of the non‐ice or snow terrestrial land as low, and 21% of the Earth's surface as remaining in a very low human influence state (Figure 3; Table S3). Independently, these datasets predict that approximately half of the planet has low human influence, and a quarter is very low influence.

We note the input data layers used in the four human influence datasets contain underlying assumptions and sources of error that, in turn, affect the level of agreement in the very low and low human influence congruence maps. Some of the human stressor datasets directly map physical sources of human activities and land use (e.g., roads and other infrastructure). Others are classified from remotely sensed imagery that have some amount of built‐in inaccuracy due to the modeling approach. For example, the ESA CCI Land Cover map has a reported 75% overall validation accuracy (Defourny et al., 2017), and accuracy assessments are only as precise as their method and validation data (Foody, 2002). Furthermore, some land cover types are easier to classify, such as built‐up areas, and there is a strong negative relationship between accuracy and landscape heterogeneity (Herold, Mayaux, Woodcock, Baccini, & Schmullius, 2008). Other studies show that certain tropical biomes can be more difficult to classify than their temperate counterparts (Jacobson et al., 2015; Tchuenté, Roujean, & de Jong, 2011). Similarly, we find that tropical and montane grassland (at the very low human influence threshold) and tropical coniferous forest (at the low threshold) biomes have the greatest classification disagreement among the four datasets (Table 5). In addition, some human stressor input data rely completely on modeling human pressure based on census data and government records (e.g., human population and livestock density). Errors here could derive from either the census data or the models used to create the spatial outputs. Problematically, any source of error in any input dataset will be carried through to the final congruency map. Thus, unanimous agreement in the congruency maps represent low human influence lands where there is a high level of confidence in this classification, but we recognize that disagreement could be due to error in a single human stressor input layer, used by a single human influence dataset.

Disagreement between the four human influence datasets compared here could also result, not only from differences in the human stressor input layers and their attributes (e.g., resolution or source year; Table S1) but also from differences in the methodology of weighting and combining these data layers (Table 2). Global Human Modification and Human Footprint combined stressors to create cumulative scores that produced continuous and ordinal datasets, respectively, whereas classification was used for Anthromes and Low Impact Areas, resulting in categorical maps. Although each dataset provides a metric for the extent of human influence on landscapes, their definitions vary, reflecting differential purposes in their creation (Table 2). Disagreement between these datasets could stem from any of these human stressor input and methodological differences, making it striking that they map similar proportions of low and very low human influence across the terrestrial surface of the planet. At the same time, there can be substantial differences at the biome scale, with large areas of spatial disagreement for certain biomes, such as temperate coniferous forests, tropical dry forests, temperate grasslands and mangroves (Table S2; Table 5).

For the purposes of this study, we focused on evaluating the agreement among four global datasets that map low or very low human influence at the broad biome scale, excluding an analysis of the extent of no direct human influence. It is likely that agreement at the level of areas completely free from human influence is lower than the figures we find here. For instance, the Human Footprint maps roughly 19% of Earth's non‐Antarctic land areas as totally free from human influence (Watson, Shanahan, et al., 2016), whereas the Human Modification maps ~5% as free from human modification (Kennedy et al., 2019). Assessments of the accuracy of these layers identify the difficulty of mapping this level of influence (Kennedy, Oakleaf, Baruch‐Mordo, Theobald, & Kiesecker, 2020; Venter, Possingham, & Watson, 2020). Moreover, we note that a consistent evaluation of areas with no human influence was not feasible across all four datasets given that Anthromes and Low Impact Areas do not map areas free from human influence.

We also acknowledge that additional global mapping efforts exist beyond those we incorporated, including those focused on accessibility/travel time to cities (Weiss et al., 2018), human appropriation of net primary productivity (Haberl et al., 2007) and Global Land Systems (van Asselen & Verburg, 2012, 2013). The choice to include or exclude any dataset would obviously affect the outcome of this analysis. Furthermore, while our analysis only considered global datasets, other human influence maps exist at regional (e.g., Cao, Carver, & Yang, 2019; Fisher et al., 2010; McGarigal et al., 2018; Perkl, 2017; Theobald, 2013) and biome scales (e.g., Bryant, Nielson, & Tangley, 1997; Henwood, 2010; Potapov et al., 2017).

### 4.1 Advances in mapping human influence on the planet

Mapping and monitoring ecosystem condition are central to monitoring progress towards most Aichi Targets (CBD & UNEP, 2010; Watson et al., 2020), the identification of the IUCN Red List of Ecosystems (Keith et al., 2013; Rodríguez et al., 2011), assessments of High Conservation Value (Brown et al., 2013) and tracking feasibility and progress towards area‐based protection targets, such as Nature Needs Half or Half‐Earth (Locke, 2013; Wilson, 2016). However, ecosystem condition has multiple dimensions including connectivity and fragmentation, genetic, species and community composition, and functional diversity. Many current approaches to mapping ecosystem condition, including the datasets in our analysis, focus on the mapping of easier‐to‐document human stressors and do not directly measure the condition of biodiversity itself (Beyer, Venter, Grantham, & Watson, 2019). These approaches can relate to important aspects of ecological condition, such as fragmentation and connectivity (Jacobson et al., 2019; Kennedy et al., 2019) or species extinction (Di Marco et al., 2018). In addition, not all aspects of biodiversity condition can be measured remotely (e.g., hunting rates or fuel gathering), thus maps of human impacts are important proxies (see O’Connor et al., 2015). The assumption is that ecosystem condition can be inferred by proxy through assessing the variety and intensity of stressors, and that ecosystems respond consistently in an empirically similar way to pressures (but see Halpern et al., 2008). However, there are a number of reasons this may not be true, for example, when stressors are not known or correctly mapped; when stressors interact with one another and with biota in unknown ways (Crain, Kroeker, & Halpern, 2008; Darling & Côté, 2008); and when ecosystems are variously resilient to stressors (Erb et al., 2017). Thus, we argue that directly measuring ecosystem condition and integrating this with advances in cumulative human pressure mapping directly are valuable approaches for the goals listed above.

Measuring and mapping condition is difficult, although progress has been made using very high‐resolution, hyperspectral and LiDAR data (Nagendra et al., 2013; Pettorelli, Owen, & Duncan, 2016). There is a limited set of studies mapping ecosystem condition from remotely sensed data, and most are local or focused on a particular biome, such as forest (e.g., Kent, Lindsell, Laurin, Valentini, & Coomes, 2015). An example of a global approach to mapping one aspect of ecosystem condition is the Biodiversity Intactness Index (Newbold et al., 2016). This produced a spatially explicit global estimate of how land use pressures have impacted species richness and abundance relative to the richness and abundance of originally present species (but see Martin, Green, & Balmford, 2019).

With continuing advances in the generation of large training datasets by citizen scientists and computer vision algorithms to analyze global‐scale satellite imagery, a new map of global ecosystem condition may be within reach (Watson et al., 2020). Commercial cloud computing platforms enable processing and analysis at global scale (Gorelick et al., 2017), and deep learning computer vision algorithms are being applied to satellite imagery analysis and monitoring on a daily basis (Finer et al., 2018). There is an opportunity to teach crowds of non‐experts to construct open‐access labeled image datasets, of sufficient scale to train algorithms that can advance automated global‐scale ecosystem condition mapping. Indeed, projects such as Geo‐Wiki (Fritz et al., 2009) and Collect Earth (Bey et al., 2016) provide platforms to use crowdsourced training data to validate land cover (Fritz et al., 2012) or develop cropland (Fritz et al., 2015) and wilderness maps (See et al., 2015). These efforts, refined to use a well‐designed, probability‐based global survey, can help direct citizen science sourcing of validation data to ensure that the maximum statistical information can be extracted from these hard‐won data (Olofsson et al., 2012; Theobald, 2016). However, image interpretation and remotely sensed assessments are likely to miss many land uses that require ground‐based assessment such as hunting, foraging and fuel‐gathering, as well as to capture land use intensity (Erb et al., 2013, 2017).

### 4.2 Conservation implications and conclusion

By combining four global maps of human influence (Anthromes, Global Human Modification, Human Footprint and Low Impact Areas), we identify the location and proportion of the planet that has relatively low human influence. Our findings suggest that ~50% of the terrestrial surface of the planet experiences low human influence and, as a consequence, it is possible to achieve bold global calls to proactively conserve at least 50% of the terrestrial planet (Baillie & Zhang, 2018; Dinerstein et al., 2017; Locke, 2013; Maron, Simmonds, & Watson, 2018; Wilson, 2016). This fits within bold protected area goals being proposed (e.g., Dinerstein et al., 2019) and also calls to proactively retain intact ecosystems via all conservation mechanisms available (e.g., Maron et al., 2018). However, as the current international conservation targets (CBD & UNEP, 2010) include clear directions for achieving ecosystem representation and connectivity and for targeting sites that are essential for achieving biodiversity conservation outcomes, it is not simply the amount but also the location of new protected areas that matters (Pimm et al., 2018; Pouzols et al., 2014; Watson, Venter, et al., 2018). We found that only two biomes (tundra and boreal forests) have a majority of datasets agreeing that at least half of their area has very low human influence, while deserts and temperate coniferous forests meet that metric at the low influence threshold (Figure 3; Table S4).

With substantial portions of low and very low influence areas found in cold and/or dry biomes, meeting full‐representational targets will be difficult (Dinerstein et al., 2017; Jacobson et al., 2019; Kennedy et al., 2019; Watson, Jones, et al., 2016). Thus, bold conservation agendas (such as trying to conserve 50% of the biome) will require conservation targets that include restoration activity (Mappin et al., 2019; Maron et al., 2018). While targeted restoration of lands with lower human influence may meet much of this shortfall, in regions where the majority of lands have been heavily modified by humans, innovative conservation solutions, such as payment for ecosystem services will be necessary (Bullock, Aronson, Newton, Pywell, & Rey‐Benayas, 2011; Mappin et al., 2019; Watson, Evans, et al., 2018). Finally, while there is a desire to conserve intact ecosystems for its own sake, there are other, potentially competing, conservation objectives that must be balanced, such as the protection of non‐intact Indigenous lands (Garnett et al., 2018), biodiverse areas that occur beyond intact ecosystems (Myers, Mittermeier, Mittermeier, Da Fonseca, & Kent, 2000; Pimm et al., 2018), degraded regions with high ecosystem services such as carbon storage capacity (Kennedy et al., 2020), or agricultural production (Mehrabi, Ellis, & Ramankutty, 2018). As such, a balanced conservation response that addresses land sovereignty, weights trade‐offs with land demands for agriculture, settlement and other resource needs (Ellis, 2019; Kennedy et al., 2016), and that speaks to the condition of Earth is essential (Locke et al., 2019). Therefore, while we illustrate the capability and strongly endorse the need to greatly increase global conservation targets, a simplistic focus on meeting area‐based targets alone is inadequate and more nuanced targets are also needed.

## Supporting information

Fig S1

Fig S2

Table S1

Table S2

Table S3
Table S4

## Data Availability

The data used in this study are openly available in DANS at https://easy.dans.knaw.nl/ui/datasets/id/easy‐dataset:74467 (Anthromes), figshare at https://doi.org/10.6084/m9.figshare.7283087.v1 (Global Human Modification), and Dryad at https://doi.org/10.5061/dryad.052q5 (Human Footprint) and https://doi.org/10.5061/dryad.z612jm67g (Low Impact Areas). Resulting overlay data are openly available on Dryad at https://doi.org/10.25338/B80G7Z.

## References

[gcb15109-bib-0001] Allan, J. R. , Venter, O. , Maxwell, S. L. , Bertzky, B. , Jones, K. R. , Shi, Y. , & Watson, J. E. M. (2017). Recent increases in human pressure and forest loss threaten many Natural World Heritage Sites. Biological Conservation, 206, 47–55. 10.1016/j.biocon.2016.12.011

[gcb15109-bib-0002] Baillie, J. E. M. , & Zhang, Y.‐P. (2018). Space for nature. Science, 361(6407), 1051 10.1126/science.aau1397 30213888

[gcb15109-bib-0003] Baldock, K. C. R. , Goddard, M. A. , Hicks, D. M. , Kunin, W. E. , Mitschunas, N. , Morse, H. , … Memmott, J. (2019). A systems approach reveals urban pollinator hotspots and conservation opportunities. Nature Ecology and Evolution, 3(3), 363–373. 10.1038/s41559-018-0769-y 30643247 PMC6445365

[gcb15109-bib-0004] Bey, A. , Sánchez‐Paus Díaz, A. , Maniatis, D. , Marchi, G. , Mollicone, D. , Ricci, S. , … Miceli, G. (2016). Collect earth: Land use and land cover assessment through augmented visual interpretation. Remote Sensing, 8(10), 807 10.3390/rs8100807

[gcb15109-bib-0005] Beyer, H. L. , Venter, O. , Grantham, H. S. , & Watson, J. E. M. (2019). Substantial losses in ecoregion intactness highlight urgency of globally coordinated action. Conservation Letters, 10.1111/conl.12692

[gcb15109-bib-0006] Brown, E. , Dudley, N. , Lindhe, A. , Mhutaman, D. R. , Stewart, C. , & Synnott, T. (2013). Common guidance for the identification of high conservation values. Retrieved from https://hcvnetwork.org/library/common‐guidance‐for‐the‐identification‐of‐high‐conservation‐values/

[gcb15109-bib-0007] Bryant, D. , Nielson, D. , & Tangley, L. (1997). The last frontier forests. Issues in Science and Technology, 14(2), 85–87. Retrieved from https://search.proquest.com/docview/195915634?pq‐origsite=gscholar

[gcb15109-bib-0008] Bullock, J. M. , Aronson, J. , Newton, A. C. , Pywell, R. F. , & Rey‐Benayas, J. M. (2011). Restoration of ecosystem services and biodiversity: Conflicts and opportunities. Trends in Ecology & Evolution, 26(10), 541–549. 10.1016/J.TREE.2011.06.011 21782273

[gcb15109-bib-0009] Cao, Y. , Carver, S. , & Yang, R. (2019). Mapping wilderness in China: Comparing and integrating Boolean and WLC approaches. Landscape and Urban Planning, 192, 103636 10.1016/j.landurbplan.2019.103636

[gcb15109-bib-0010] CBD, & UNEP . (2010). Strategic plan for biodiversity 2011–2020 and the Aichi targets. Montreal, Canada Retrieved from http://www.cbd.int/doc/strategic‐plan/2011–2020/Aichi‐Targets‐EN.pdf

[gcb15109-bib-0011] Crain, C. M. , Kroeker, K. , & Halpern, B. S. (2008). Interactive and cumulative effects of multiple human stressors in marine systems. Ecology Letters, 11(12), 1304–1315. 10.1111/j.1461-0248.2008.01253.x 19046359

[gcb15109-bib-0012] Darling, E. S. , & Côté, I. M. (2008). Quantifying the evidence for ecological synergies. Ecology Letters, 11(12), 1278–1286. 10.1111/j.1461-0248.2008.01243.x 18785986

[gcb15109-bib-0013] Defourny, P. , Santoro, M. , Kirches, G. , Wevers, J. , Boettcher, M. , Brockmann, C. , & Moreau, I. (2017). Land cover CCI: Product user guide version 2.0. Retrieved from http://maps.elie.ucl.ac.be/CCI/viewer/download/ESACCI‐LC‐PUG‐v2.5.pdf

[gcb15109-bib-0014] Di Marco, M. , Ferrier, S. , Harwood, T. D. , Hoskins, A. J. , & Watson, J. E. M. (2019). Wilderness areas halve the extinction risk of terrestrial biodiversity. Nature, 573(7775), 582–585. 10.1038/s41586-019-1567-7 31534225

[gcb15109-bib-0015] Di Marco, M. , Venter, O. , Possingham, H. P. , & Watson, J. E. M. (2018). Changes in human footprint drive changes in species extinction risk. Nature Communications, 9(1), 4621 10.1038/s41467-018-07049-5 PMC6218474 30397204

[gcb15109-bib-0016] Díaz, S. , Pascual, U. , Stenseke, M. , Martín‐López, B. , Watson, R. T. , Molnár, Z. , … Shirayama, Y. (2018). Assessing nature’s contributions to people. Science, 359(6373), 270–272. 10.1126/science.aap8826 29348221

[gcb15109-bib-0017] Dinerstein, E. , Olson, D. , Joshi, A. , Vynne, C. , Burgess, N. D. , Wikramanayake, E. , … Saleem, M. (2017). An ecoregion‐based approach to protecting half the terrestrial realm. BioScience, 67(6), 534–545. 10.1093/biosci/bix014 28608869 PMC5451287

[gcb15109-bib-0018] Dinerstein, E. , Vynne, C. , Sala, E. , Joshi, A. R. , Fernando, S. , Lovejoy, T. E. , … Wikramanayake, E. (2019). A global deal for nature: Guiding principles, milestones, and targets. Science Advances, 5(4), eaaw2869 10.1126/sciadv.aaw2869 31016243 PMC6474764

[gcb15109-bib-0019] Edwards, D. P. , Tobias, J. A. , Sheil, D. , Meijaard, E. , & Laurance, W. F. (2014). Maintaining ecosystem function and services in logged tropical forests. Trends in Ecology & Evolution, 29(9), 511–520. 10.1016/j.tree.2014.07.003 25092495

[gcb15109-bib-0020] Ellis, E. C. (2019). To conserve nature in the Anthropocene, half earth is not nearly enough. One Earth, 1(2), 163–167. 10.1016/j.oneear.2019.10.009

[gcb15109-bib-0021] Ellis, E. C. , Goldewijk, K. K. , Siebert, S. , Lightman, D. , & Ramankutty, N. (2010). Anthropogenic transformation of the biomes, 1700 to 2000. Global Ecology and Biogeography, 19(5), 589–606. 10.1111/j.1466-8238.2010.00540.x

[gcb15109-bib-0022] Ellis, E. C. , & Ramankutty, N. (2008). Putting people in the map: Anthropogenic biomes of the world. Frontiers in Ecology and the Environment, 6(8), 439–447. 10.1890/070062

[gcb15109-bib-0023] Erb, K.‐H. , Haberl, H. , Jepsen, M. R. , Kuemmerle, T. , Lindner, M. , Müller, D. , … Reenberg, A. (2013). A conceptual framework for analysing and measuring land‐use intensity. Current Opinion in Environmental Sustainability, 5(5), 464–470. 10.1016/j.cosust.2013.07.010 24143156 PMC3798045

[gcb15109-bib-0024] Erb, K.‐H. , Luyssaert, S. , Meyfroidt, P. , Pongratz, J. , Don, A. , Kloster, S. , … Dolman, A. J. (2017). Land management: Data availability and process understanding for global change studies. Global Change Biology, 23(2), 512–533. 10.1111/gcb.13443 27447350

[gcb15109-bib-0025] Ewert, A. , Overholt, J. , Voight, A. , & Wang, C. C. (2011). Understanding the transformative aspects of the wilderness and protected lands experience upon human health In Watson A., Murrieta‐Saldivar J., & McBride B. (Eds.), Science and stewardship to protect and sustain wilderness values: Ninth World Wilderness Congress symposium; November 6–13, 2009; Merida, Yucatan, Mexico (pp. 140–146). Fort Collins, CO: U.S. Department of Agriculture, Forest Service, Rocky Mountain Research Station.

[gcb15109-bib-0026] Finer, M. , Novoa, S. , Weisse, M. J. , Petersen, R. , Mascaro, J. , Souto, T. , … Martinez, R. G. (2018). Combating deforestation: From satellite to intervention. Science, 360(6395), 1303–1305. 10.1126/science.aat1203 29930127

[gcb15109-bib-0027] Fisher, M. , Carver, S. , Kun, Z. , McMorran, R. , Arrell, K. , Mitchell, G. , & Kun, S. (2010). Review of status and conservation of wild land in Europe. The Wildland Research Institute Retrieved from https://wildlandresearch.org/downloads/publications/

[gcb15109-bib-0028] Foody, G. M. (2002). Status of land cover classification accuracy assessment. Remote Sensing of Environment, 80(1), 185–201. 10.1016/S0034-4257(01)00295-4

[gcb15109-bib-0029] Fritz, S. , McCallum, I. , Schill, C. , Perger, C. , Grillmayer, R. , Achard, F. , … Obersteiner, M. (2009). Geo‐wiki.org: The use of crowdsourcing to improve global land cover. Remote Sensing, 1(3), 345–354. 10.3390/rs1030345

[gcb15109-bib-0030] Fritz, S. , McCallum, I. , Schill, C. , Perger, C. , See, L. , Schepaschenko, D. , … Obersteiner, M. (2012). Geo‐Wiki: An online platform for improving global land cover. Environmental Modelling and Software, 31, 110–123. 10.1016/j.envsoft.2011.11.015

[gcb15109-bib-0031] Fritz, S. , See, L. , McCallum, I. , You, L. , Bun, A. , Moltchanova, E. , … Obersteiner, M. (2015). Mapping global cropland and field size. Global Change Biology, 21(5), 1980–1992. 10.1111/gcb.12838 25640302

[gcb15109-bib-0032] Garnett, S. T. , Burgess, N. D. , Fa, J. E. , Fernández‐Llamazares, Á. , Molnár, Z. , Robinson, C. J. , … Leiper, I. (2018). A spatial overview of the global importance of Indigenous lands for conservation. Nature Sustainability, 1(7), 369–374. 10.1038/s41893-018-0100-6

[gcb15109-bib-0033] Gibbs, H. K. , & Salmon, J. M. (2015). Mapping the world’s degraded lands. Applied Geography, 57, 12–21. 10.1016/j.apgeog.2014.11.024

[gcb15109-bib-0034] Goldewijk, K. K. , Beusen, A. , Doelman, J. , & Stehfest, E. (2017). Anthropogenic land use estimates for the Holocene – HYDE 3.2. Earth System Science Data, 9(2), 927–953. 10.5194/essd-9-927-2017

[gcb15109-bib-0035] Gorelick, N. , Hancher, M. , Dixon, M. , Ilyushchenko, S. , Thau, D. , & Moore, R. (2017). Google earth engine: Planetary‐scale geospatial analysis for everyone. Remote Sensing of Environment, 202, 18–27. 10.1016/j.rse.2017.06.031

[gcb15109-bib-0036] Green, R. E. , Cornell, S. J. , Scharlemann, J. P. W. , & Balmford, A. (2005). Farming and the fate of wild nature. Science, 307(5709), 550–555. 10.1126/science.1106049 15618485

[gcb15109-bib-0037] Haberl, H. , Erb, K. H. , Krausmann, F. , Gaube, V. , Bondeau, A. , Plutzar, C. , … Fischer‐Kowalski, M. (2007). Quantifying and mapping the human appropriation of net primary production in earth’s terrestrial ecosystems. Proceedings of the National Academy of Sciences of the United States of America, 104(31), 12942–12947. 10.1073/pnas.0704243104 17616580 PMC1911196

[gcb15109-bib-0038] Halpern, B. S. , Walbridge, S. , Selkoe, K. A. , Kappel, C. V. , Micheli, F. , D'Agrosa, C. , … Watson, R. (2008). A global map of human impact on marine ecosystems. Science, 319(5865), 948–952. 10.1126/science.1149345 18276889

[gcb15109-bib-0039] Hannah, L. , Lohse, D. , Hutchinson, C. , Carr, J. L. , & Lankerani, A. (1994). A preliminary inventory of human disturbance of world ecosystems. Ambio, 23(4–5), 246–250. 10.1016/0006-3207(96)83209-5

[gcb15109-bib-0040] Henwood, W. D. (2010). Toward a strategy for the conservation and protection of the world’s temperate grasslands. Great Plains Research, 20(1), 121–134.

[gcb15109-bib-0041] Herold, M. , Mayaux, P. , Woodcock, C. E. , Baccini, A. , & Schmullius, C. (2008). Some challenges in global land cover mapping: An assessment of agreement and accuracy in existing 1 km datasets. Remote Sensing of Environment, 112(5), 2538–2556. 10.1016/j.rse.2007.11.013

[gcb15109-bib-0042] Jaccard, P. (1912). The distribution of the flora in the alpine zone. New Phytologist, 11(2), 37–50. 10.1111/j.1469-8137.1912.tb05611.x

[gcb15109-bib-0043] Jacobson, A. , Dhanota, J. , Godfrey, J. , Jacobson, H. , Rossman, Z. , Stanish, A. , … Riggio, J. (2015). A novel approach to mapping land conversion using Google Earth with an application to East Africa. Environmental Modelling & Software, 72, 1–9. 10.1016/j.envsoft.2015.06.011

[gcb15109-bib-0044] Jacobson, A. P. , Riggio, J. , Tait, A. M. , & Baillie, J. E. M. (2019). Global areas of low human impact (‘Low Impact Areas’) and fragmentation of the natural world. Scientific Reports, 9(1), 14179 10.1038/s41598-019-50558-6 31578431 PMC6775135

[gcb15109-bib-0045] Jones, K. R. , Venter, O. , Fuller, R. A. , Allan, J. R. , Maxwell, S. L. , Negret, P. J. , & Watson, J. E. M. (2018). One‐third of global protected land is under intense human pressure. Science, 360(6390), 788–791. 10.1126/science.aap9565 29773750

[gcb15109-bib-0046] Keith, D. A. , Rodríguez, J. P. , Rodríguez‐Clark, K. M. , Nicholson, E. , Aapala, K. , Alonso, A. , … Zambrano‐Martínez, S. (2013). Scientific foundations for an IUCN red list of ecosystems. PLoS ONE, 8(5), e62111 10.1371/journal.pone.0062111 23667454 PMC3648534

[gcb15109-bib-0047] Kennedy, C. M. , Hawthorne, P. L. , Miteva, D. A. , Baumgarten, L. , Sochi, K. , Matsumoto, M. , … Kiesecker, J. (2016). Optimizing land use decision‐making to sustain Brazilian agricultural profits, biodiversity and ecosystem services. Biological Conservation, 204, 221–230. 10.1016/j.biocon.2016.10.039

[gcb15109-bib-0048] Kennedy, C. M. , Oakleaf, J. R. , Baruch‐Mordo, S. , Theobald, D. M. , & Kiesecker, J. (2020). Finding middle ground: Extending conservation beyond wilderness areas. Global Change Biology, 26(2), 333–336. 10.1111/gcb.14900 31674120

[gcb15109-bib-0049] Kennedy, C. M. , Oakleaf, J. R. , Theobald, D. M. , Baruch‐Mordo, S. , & Kiesecker, J. (2018). Global Human Modification. 10.6084/m9.figshare.7283087 30629311

[gcb15109-bib-0050] Kennedy, C. M. , Oakleaf, J. R. , Theobald, D. M. , Baruch‐Mordo, S. , & Kiesecker, J. (2019). Managing the middle: A shift in conservation priorities based on the global human modification gradient. Global Change Biology, 25(3), 811–826. 10.1111/gcb.14549 30629311

[gcb15109-bib-0051] Kent, R. , Lindsell, J. A. , Laurin, G. V. , Valentini, R. , & Coomes, D. A. (2015). Airborne LiDAR detects selectively logged tropical forest even in an advanced stage of recovery. Remote Sensing, 7(7), 8348–8367. 10.3390/rs70708348

[gcb15109-bib-0052] Klein Goldewijk, K. , & Ramankutty, N. (2004). Land cover change over the last three centuries due to human activities: The availability of new global data sets. GeoJournal, 61(4), 335–344. 10.1007/s10708-004-5050-z

[gcb15109-bib-0053] Krausmann, F. , Erb, K.‐H. , Gingrich, S. , Haberl, H. , Bondeau, A. , Gaube, V. , … Searchinger, T. D. (2013). Global human appropriation of net primary production doubled in the 20th century. Proceedings of the National Academy of Sciences of the United States of America, 110(25), 10324–10329. 10.1073/pnas.1211349110 23733940 PMC3690849

[gcb15109-bib-0054] Kremen, C. , & Merenlender, A. M. (2018). Landscapes that work for biodiversity and people. Science, 362(6412), eaau6020 10.1126/science.aau6020 30337381

[gcb15109-bib-0055] Kühl, H. S. , Boesch, C. , Kulik, L. , Haas, F. , Arandjelovic, M. , Dieguez, P. , … Kalan, A. K. (2019). Human impact erodes chimpanzee behavioral diversity. Science, 363(6434), 1453–1455. 10.1126/science.aau4532 30846610

[gcb15109-bib-0056] Lesslie, R. G. (1998). Global wilderness. Cambridge, UK: UNEP‐WCMC.

[gcb15109-bib-0057] Levandowsky, M. , & Winter, D. (1971). Distance between sets. Nature, 234(5323), 34–35. 10.1038/234034a0

[gcb15109-bib-0058] Locke, H. (2013). Nature needs half: A necessary and hopeful new agenda for protected areas. Parks, 19(2), 13–22. 10.2305/IUCN.CH.2013.PARKS-19-2.HL.en

[gcb15109-bib-0059] Locke, H. , Ellis, E. C. , Venter, O. , Schuster, R. , Ma, K. , Shen, X. , … Watson, J. E. M. (2019). Three global conditions for biodiversity conservation and sustainable use: An implementation framework. National Science Review, 6(6), 1080–1082. 10.1093/nsr/nwz136 PMC8291457 34691979

[gcb15109-bib-0060] Mappin, B. , Chauvenet, A. L. M. , Adams, V. M. , Di Marco, M. , Beyer, H. L. , Venter, O. , … Watson, J. E. M. (2019). Restoration priorities to achieve the global protected area target. Conservation Letters, 12(4), e12646 10.1111/conl.12646

[gcb15109-bib-0061] Maron, M. , Simmonds, J. S. , & Watson, J. E. M. (2018). Bold nature retention targets are essential for the global environment agenda. Nature Ecology & Evolution, 2(8), 1194–1195. 10.1038/s41559-018-0595-2 29915340

[gcb15109-bib-0062] Martin, P. A. , Green, R. E. , & Balmford, A. (2019). The biodiversity intactness index may underestimate losses. Nature Ecology & Evolution, 3(6), 862–863. 10.1038/s41559-019-0895-1 31061478

[gcb15109-bib-0063] Martin, T. G. , & Watson, J. E. M. (2016). Intact ecosystems provide best defence against climate change. Nature Climate Change, 6(2), 122–124. 10.1038/nclimate2918

[gcb15109-bib-0064] McCloskey, J. M. , & Spalding, H. (1989). A reconnaissance‐level inventory of the amount of wilderness remaining in the world. Ambio, 18(4), 221–227. 10.2307/4313570

[gcb15109-bib-0065] McGarigal, K. , Compton, B. W. , Plunkett, E. B. , DeLuca, W. V. , Grand, J. , Ene, E. , & Jackson, S. D. (2018). A landscape index of ecological integrity to inform landscape conservation. Landscape Ecology, 33(7), 1029–1048. 10.1007/s10980-018-0653-9

[gcb15109-bib-0066] Mehrabi, Z. , Ellis, E. C. , & Ramankutty, N. (2018). The challenge of feeding the world while conserving half the planet. Nature Sustainability, 1(8), 409–412. 10.1038/s41893-018-0119-8

[gcb15109-bib-0067] Mittermeier, R. A. , Mittermeier, C. G. , Gil, P. R. , Pilgrim, J. D. , Fonseca, G. , Brooks, T. M. , & Konstant, W. R. (2002). Wilderness: Earth’s last wild places. Mexico: CEMEX.

[gcb15109-bib-0068] Myers, N. , Mittermeier, R. A. , Mittermeier, C. G. , Da Fonseca, G. A. B. , & Kent, J. (2000). Biodiversity hotspots for conservation priorities. Nature, 403(6772), 853–858. 10.1038/35002501 10706275

[gcb15109-bib-0069] Nagendra, H. , Lucas, R. , Honrado, J. P. , Jongman, R. H. G. , Tarantino, C. , Adamo, M. , & Mairota, P. (2013). Remote sensing for conservation monitoring: Assessing protected areas, habitat extent, habitat condition, species diversity, and threats. Ecological Indicators, 33, 45–59. 10.1016/j.ecolind.2012.09.014

[gcb15109-bib-0070] Newbold, T. , Hudson, L. N. , Arnell, A. P. , Contu, S. , De Palma, A. , Ferrier, S. , … Purvis, A. (2016). Has land use pushed terrestrial biodiversity beyond the planetary boundary? A global assessment. Science, 353(6296), 288–291. 10.1126/science.aaf2201 27418509

[gcb15109-bib-0071] O’Connor, B. , Secades, C. , Penner, J. , Sonnenschein, R. , Skidmore, A. K. , Burgess, N. D. , & Hutton, J. M. (2015). Earth observation as a tool for tracking progress towards the Aichi Biodiversity Targets. Remote Sensing in Ecology and Conservation, 1(1), 19–28. 10.1002/rse2.4

[gcb15109-bib-0072] Oakleaf, J. R. , Kennedy, C. M. , Baruch‐Mordo, S. , West, P. C. , Gerber, J. S. , Jarvis, L. , & Kiesecker, J. (2015). A world at risk: Aggregating development trends to forecast global habitat conversion. PLoS ONE, 10(10), e0138334 10.1371/journal.pone.0138334 26445282 PMC4596827

[gcb15109-bib-0073] Olofsson, P. , Stehman, S. V. , Woodcock, C. E. , Sulla‐Menashe, D. , Sibley, A. M. , Newell, J. D. , … Herold, M. (2012). A global land‐cover validation data set, part I: Fundamental design principles. International Journal of Remote Sensing, 33(18), 5768–5788. 10.1080/01431161.2012.674230

[gcb15109-bib-0074] Perkl, R. M. (2017). Measuring landscape integrity (LI): Development of a hybrid methodology for planning applications. Journal of Environmental Planning and Management, 60(1), 92–114. 10.1080/09640568.2016.1142863

[gcb15109-bib-0075] Pettorelli, N. , Owen, H. J. F. , & Duncan, C. (2016). How do we want Satellite Remote Sensing to support biodiversity conservation globally? Methods in Ecology and Evolution, 7(6), 656–665. 10.1111/2041-210X.12545

[gcb15109-bib-0076] Pimm, S. L. , Jenkins, C. N. , & Li, B. V. (2018). How to protect half of Earth to ensure it protects sufficient biodiversity. Science Advances, 4(8), eaat2616 10.1126/sciadv.aat2616 30167461 PMC6114985

[gcb15109-bib-0077] Potapov, P. , Hansen, M. C. , Laestadius, L. , Turubanova, S. , Yaroshenko, A. , Thies, C. , … Esipova, E. (2017). The last frontiers of wilderness: Tracking loss of intact forest landscapes from 2000 to 2013. Science Advances, 3(1), e1600821 10.1126/sciadv.1600821 28097216 PMC5235335

[gcb15109-bib-0078] Potere, D. , & Schneider, A. (2007). A critical look at representations of urban areas in global maps. GeoJournal, 69(1–2), 55–80. 10.1007/s10708-007-9102-z

[gcb15109-bib-0079] Pouzols, F. M. , Toivonen, T. , Di Minin, E. , Kukkala, A. S. , Kullberg, P. , Kuusterä, J. , … Moilanen, A. (2014). Global protected area expansion is compromised by projected land‐use and parochialism. Nature, 516(7531), 383–386. 10.1038/nature14032 25494203

[gcb15109-bib-0080] Rodríguez, J. P. , Rodríguez‐Clark, K. M. , Baillie, J. E. M. , Ash, N. , Benson, J. , Boucher, T. , … Zamin, T. (2011). Establishing IUCN Red List criteria for threatened ecosystems. Conservation Biology, 25(1), 21–29. 10.1111/j.1523-1739.2010.01598.x 21054525 PMC3051828

[gcb15109-bib-0081] Sanderson, E. W. , Jaiteh, M. , Levy, M. A. , Redford, K. H. , Wannebo, A. V. , & Woolmer, G. (2002). The human footprint and the last of the wild: The human footprint is a global map of human influence on the land surface, which suggests that human beings are stewards of nature, whether we like it or not. BioScience, 52(10), 891–904. 10.1641/0006-3568(2002)052[0891:THFATL]2.0.CO;2

[gcb15109-bib-0082] See, L. , Fritz, S. , Perger, C. , Schill, C. , McCallum, I. , Schepaschenko, D. , … Obersteiner, M. (2015). Harnessing the power of volunteers, the internet and Google Earth to collect and validate global spatial information using Geo‐Wiki. Technological Forecasting and Social Change, 98, 324–335. 10.1016/j.techfore.2015.03.002

[gcb15109-bib-0083] Tchuenté, A. T. K. , Roujean, J. L. , & de Jong, S. M. (2011). Comparison and relative quality assessment of the GLC2000, GLOBCOVER, MODIS and ECOCLIMAP land cover data sets at the African continental scale. International Journal of Applied Earth Observation and Geoinformation, 13(2), 207–219. 10.1016/j.jag.2010.11.005

[gcb15109-bib-0084] Theobald, D. M. (2013). A general model to quantify ecological integrity for landscape assessments and US application. Landscape Ecology, 28(10), 1859–1874. 10.1007/s10980-013-9941-6

[gcb15109-bib-0085] Theobald, D. M. (2016). A general‐purpose spatial survey design for collaborative science and monitoring of global environmental change: The Global Grid. Remote Sensing, 8(10), 813 10.3390/rs8100813

[gcb15109-bib-0086] Tuanmu, M. N. , & Jetz, W. (2014). A global 1‐km consensus land‐cover product for biodiversity and ecosystem modelling. Global Ecology and Biogeography, 23(9), 1031–1045. 10.1111/geb.12182

[gcb15109-bib-0087] Tucker, M. A. , Böhning‐Gaese, K. , Fagan, W. F. , Fryxell, J. M. , Van Moorter, B. , Alberts, S. C. , … Mueller, T. (2018). Moving in the Anthropocene: Global reductions in terrestrial mammalian movements. Science, 359(6374), 466–469. 10.1126/science.aam9712 29371471

[gcb15109-bib-0088] UNEP‐WCMC, IUCN, & NGS . (2019). Protected planet live report 2019. Cambridge, UK; Gland, Switzerland; and Washington, DC Retrieved from https://livereport.protectedplanet.net/

[gcb15109-bib-0089] van Asselen, S. , & Verburg, P. H. (2012). A land system representation for global assessments and land‐use modeling. Global Change Biology, 18(10), 3125–3148. 10.1111/j.1365-2486.2012.02759.x 28741836

[gcb15109-bib-0090] van Asselen, S. , & Verburg, P. H. (2013). Land cover change or land‐use intensification: Simulating land system change with a global‐scale land change model. Global Change Biology, 19(12), 3648–3667. 10.1111/gcb.12331 23893426

[gcb15109-bib-0091] Venter, O. , Possingham, H. P. , & Watson, J. E. (2020). The human footprint represents observable human pressures: Reply to Kennedy et al Global Change Biology, 26(2), 330–332. 10.1111/gcb.14849 31578793

[gcb15109-bib-0092] Venter, O. , Sanderson, E. W. , Magrach, A. , Allan, J. R. , Beher, J. , Jones, K. R. , … Watson, J. E. M. (2016). Sixteen years of change in the global terrestrial human footprint and implications for biodiversity conservation. Nature Communications, 7(1), 12558 10.1038/ncomms12558 PMC4996975 27552116

[gcb15109-bib-0093] Watson, J. E. M. , Evans, T. , Venter, O. , Williams, B. , Tulloch, A. , Stewart, C. , … Lindenmayer, D. (2018). The exceptional value of intact forest ecosystems. Nature Ecology and Evolution, 2(4), 599–610. 10.1038/s41559-018-0490-x 29483681

[gcb15109-bib-0094] Watson, J. E. M. , Jones, K. R. , Fuller, R. A. , Marco, M. D. , Segan, D. B. , Butchart, S. H. M. , … Venter, O. (2016). Persistent disparities between recent rates of habitat conversion and protection and implications for future global conservation targets. Conservation Letters, 9(6), 413–421. 10.1111/conl.12295

[gcb15109-bib-0095] Watson, J. E. M. , Keith, D. A. , Strassburg, B. B. N. , Venter, O. , Williams, B. , & Nicholson, E. (2020). Set a global target for ecosystems. Nature, 578(7795), 360–362. 10.1038/d41586-020-00446-1 32071453

[gcb15109-bib-0096] Watson, J. E. M. , Shanahan, D. F. , Di Marco, M. , Allan, J. , Laurance, W. F. , Sanderson, E. W. , … Venter, O. (2016). Catastrophic declines in wilderness areas undermine global environment targets. Current Biology, 26(21), 2929–2934. 10.1016/j.cub.2016.08.049 27618267

[gcb15109-bib-0097] Watson, J. E. M. , & Venter, O. (2017). A global plan for nature conservation. Nature, 550(7674), 48–49. 10.1038/nature24144 28953876

[gcb15109-bib-0098] Watson, J. E. M. , & Venter, O. (2019). Mapping the continuum of humanity’s footprint on land. One Earth, 1(2), 175–180. 10.1016/j.oneear.2019.09.004

[gcb15109-bib-0099] Watson, J. E. M. , Venter, O. , Lee, J. , Jones, K. R. , Robinson, J. G. , Possingham, H. P. , & Allan, J. R. (2018). Protect the last of the wild. Nature, 563(7729), 27–30. 10.1038/d41586-018-07183-6 30382225

[gcb15109-bib-0100] Weiss, D. J. , Nelson, A. , Gibson, H. S. , Temperley, W. , Peedell, S. , Lieber, A. , … Gething, P. W. (2018). A global map of travel time to cities to assess inequalities in accessibility in 2015. Nature, 553(7688), 333–336. 10.1038/nature25181 29320477

[gcb15109-bib-0101] Wilson, E. O. (2016). Half‐earth: Our planet’s fight for life. New York: W.W. Norton & Company 10.1149/1.3635578

