# Supplementary figures and images for: Global human influence maps reveal clear opportunities in conserving Earth’s remaining intact terrestrial ecosystems
Source: Glob Chang Biol. 2020 Jun 5;26(8):4344–56. doi: 10.1111/gcb.15109 (PMC7383735; doi:10.1111/gcb.15109)

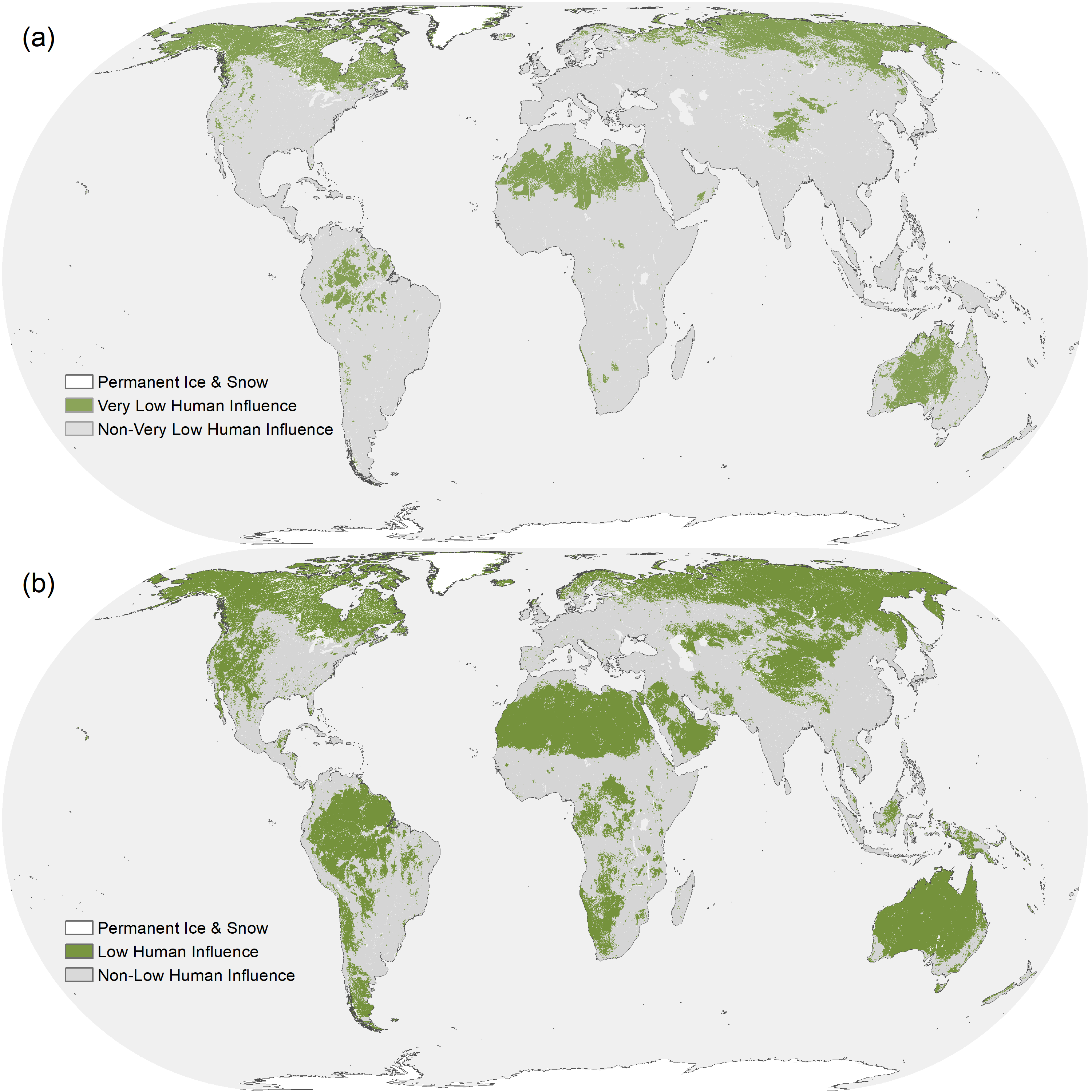

Supplement: Supplementary file 1 — Fig S1 [file GCB-26-4344-s001.tif]

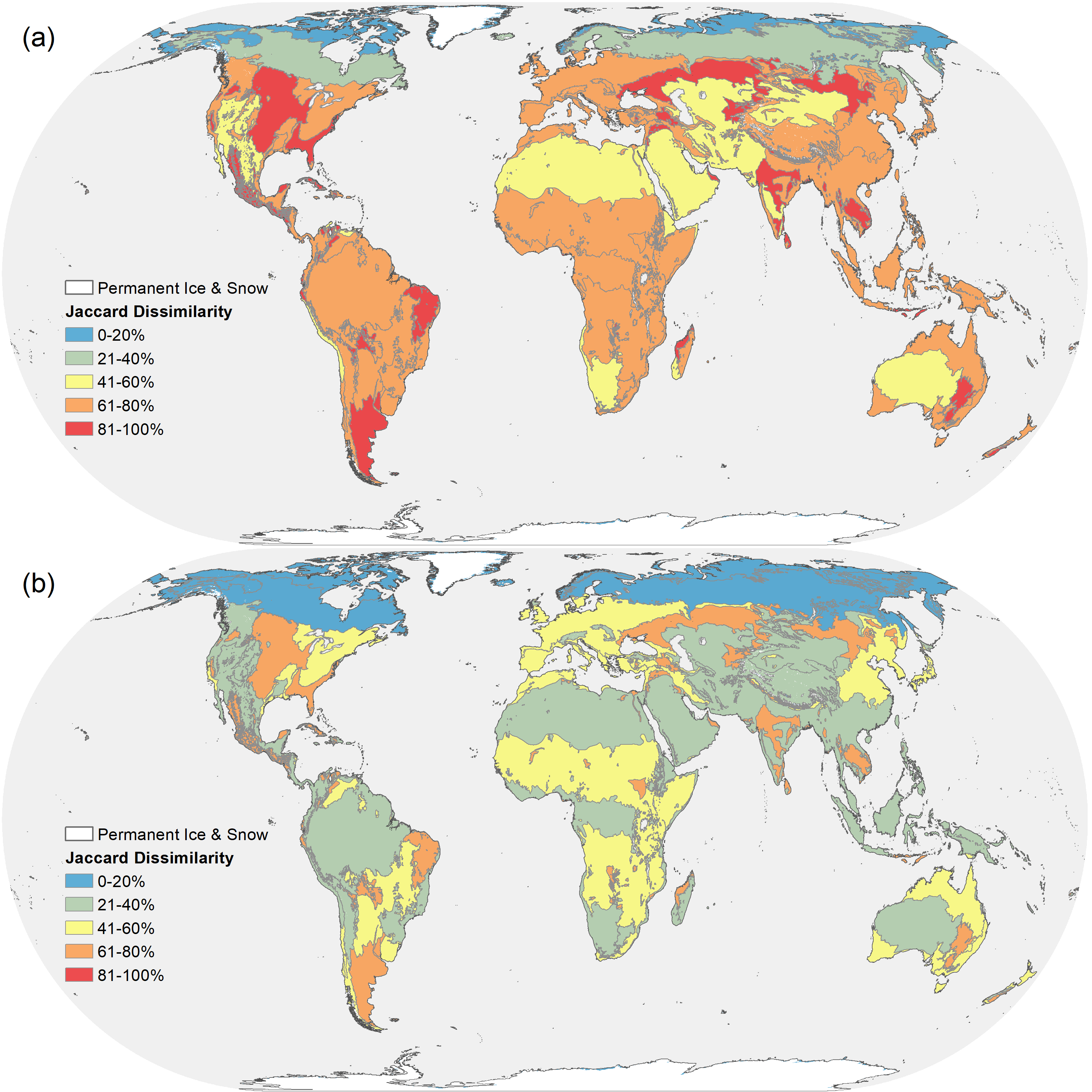

Supplement: Supplementary file 2 — Fig S2 [file GCB-26-4344-s002.tif]
