# Supplementary material for: Global human influence maps reveal clear opportunities in conserving Earth’s remaining intact terrestrial ecosystems
Source: Glob Chang Biol. 2020 Jun 5;26(8):4344–56. doi: 10.1111/gcb.15109 (PMC7383735; doi:10.1111/gcb.15109)
Supplement: Supplementary file 5 — Table S3 Table S4 [file GCB-26-4344-s005.docx]

Table S3: Overall percent agreement between the four input datasets classification of low and very low human influence regions of the world.

| **Classification Agreement** | **Very Low Influence** | **Low Influence** |
| --- | --- | --- |
| Full | 13.1% | 35.2% |
| Majority | 20.7% | 46.3% |
| Mixed | 8.5% | 9.4% |
| Minority | 70.9% | 44.3% |
| None | 59.3% | 30.4% |

Table S4: Overall percent agreement between the four input datasets classification of low and very low human influence regions of the world classified by biome (Dinerstein et al. 2017).

| **A.** | **Very Low Human Influence Classification Agreement** | | | | | |
| --- | --- | --- | --- | --- | --- | --- |
| **Biome Name** | **Full** | **Majority** | **Mixed** | **Minority** | **None** |  |
| Tundra | 78.8% | 91.5% | 4.6% | 3.9% | 1.4% |  |
| Boreal Forests | 42.5% | 60.2% | 15.1% | 24.7% | 13.0% |  |
| Deserts | 14.3% | 28.2% | 14.1% | 57.6% | 42.7% |  |
| Temperate Coniferous Forests | 6.4% | 12.7% | 14.6% | 72.6% | 54.3% |  |
| Tropical Moist Forests | 3.5% | 8.5% | 8.0% | 83.5% | 67.7% |  |
| Mediterranean | 2.4% | 4.5% | 3.6% | 91.9% | 85.1% |  |
| Flooded Grasslands | 1.5% | 2.8% | 3.1% | 94.1% | 76.7% |  |
| Temperate Broadleaf Forests | 1.2% | 2.7% | 2.9% | 94.4% | 88.4% |  |
| Tropical Grasslands | 0.9% | 4.1% | 7.1% | 88.8% | 77.7% |  |
| Mangroves | 0.6% | 1.2% | 2.9% | 95.9% | 80.7% |  |
| Montane Grasslands | 0.4% | 9.9% | 10.0% | 80.1% | 59.9% |  |
| Temperate Grasslands | 0.0% | 0.2% | 0.6% | 99.2% | 93.3% |  |
| Tropical Coniferous Forests | 0.0% | 0.1% | 0.3% | 99.6% | 95.8% |  |
| Tropical Dry Forests | 0.0% | 0.2% | 0.4% | 99.4% | 95.7% |  |
|  |  |  |  |  |  |  |
| **B.** | **Low Human Influence Classification Agreement** | | | | |  |
| **Biome Name** | **Full** | **Majority** | **Mixed** | **Minority** | **None** |  |
| Tundra | 94.6% | 98.5% | 0.9% | 0.6% | 0.1% |  |
| Boreal Forests | 77.4% | 89.4% | 5.0% | 5.6% | 2.1% |  |
| Deserts | 51.2% | 68.2% | 10.0% | 21.8% | 12.8% |  |
| Temperate Coniferous Forests | 41.1% | 58.0% | 11.1% | 31.0% | 17.5% |  |
| Montane Grasslands | 29.4% | 43.2% | 13.7% | 43.1% | 27.1% |  |
| Tropical Moist Forests | 24.8% | 34.4% | 10.9% | 54.8% | 38.3% |  |
| Tropical Grasslands | 18.3% | 29.1% | 12.5% | 58.4% | 38.3% |  |
| Flooded Grasslands | 10.5% | 20.8% | 20.0% | 59.1% | 37.9% |  |
| Mediterranean | 10.5% | 18.0% | 7.5% | 74.4% | 59.5% |  |
| Temperate Broadleaf Forests | 7.5% | 12.3% | 6.6% | 81.1% | 63.1% |  |
| Temperate Grasslands | 7.4% | 19.4% | 11.7% | 68.9% | 46.3% |  |
| Mangroves | 5.8% | 14.4% | 13.5% | 72.1% | 47.7% |  |
| Tropical Dry Forests | 2.9% | 6.7% | 7.1% | 86.2% | 69.3% |  |
| Tropical Coniferous Forests | 2.4% | 9.8% | 15.8% | 74.4% | 48.9% |  |
